# A 4-year cohort study of the effects of PNPLA3 rs738409 genotypes on liver fat and fibrosis and gut microbiota in a non-fatty liver population

**DOI:** 10.1265/ehpm.24-00365

**Published:** 2025-03-12

**Authors:** Satoshi Sato, Chikara Iino, Takafumi Sasada, Keisuke Furusawa, Kenta Yoshida, Kaori Sawada, Tatsuya Mikami, Shinsaku Fukuda, Shigeyuki Nakaji, Hirotake Sakuraba

**Affiliations:** 1 Department of Gastroenterology, Hematology and Clinical Immunology, Hirosaki University Graduate School of Medicine, Hirosaki 036-8562, Japan; 2 Department of Preemptive Medicine, Hirosaki University Graduate School of Medicine, Hirosaki 036-8562, Japan

**Keywords:** Liver fat, Liver fibrosis, Patatin-like phospholipase domain-containing 3 (PNPLA3), Gut microbiota, Cohort study

## Abstract

**Background:**

Many factors are associated with the development and progression of liver fat and fibrosis; however, genetics and the gut microbiota are representative factors. Moreover, recent studies have indicated a link between host genes and the gut microbiota. This study investigated the effect of patatin-like phospholipase domain-containing 3 (PNPLA3) rs738409 (C > G), which has been reported to be most involved in the onset and progression of fatty liver, on liver fat and fibrosis in a cohort study related to gut microbiota in a non-fatty liver population.

**Methods:**

This cohort study included 214 participants from the health check-up project in 2018 and 2022 who had non-fatty liver with controlled attenuation parameter (CAP) values <248 dB/m by FibroScan and were non-drinkers. Changes in CAP values and liver stiffness measurement (LSM), liver-related items, and gut microbiota from 2018 to 2022 were investigated separately for PNPLA3 rs738409 CC, CG, and GG genotypes.

**Results:**

Baseline values showed no difference among the PNPLA3 rs738409 genotypes for any of the measurement items. From 2018 to 2022, the PNPLA3 rs738409 CG and GG genotype groups showed a significant increase in CAP and body mass index; no significant change was observed in the CC genotype group. LSM increased in all genotypes, but the rate of increase was highest in the GG genotype, followed by the CG and CC genotypes. Fasting blood glucose levels increased in all genotypes; however, HOMA-IR (Homeostasis Model Assessment of Insulin Resistance) increased significantly only in the GG genotype. HDL (high-density lipoprotein) and LDL (low-density lipoprotein) cholesterol levels significantly increased in all genotypes, whereas triglycerides did not show any significant changes in any genotype. As for the gut microbiota, the relative abundance of *Feacalibacterium* in the PNPLA3 rs738409 GG genotype decreased by 2% over 4 years, more than 2-fold compared to CC and GG genotypes. *Blautia* increased significantly in the CC group.

**Conclusion:**

The results suggest that PNPLA3 G-allele carriers of non-fatty liver develop liver fat and fibrosis due to not only obesity and insulin resistance but also the deterioration of gut microbiota, which may require a relatively long course of time, even years.

## Background

Fatty liver is asymptomatic but can cause cardiovascular disease and hepatitis, leading to liver cirrhosis and the risk of liver cancer. Moreover, recent studies have shown that fatty liver is associated not only with obesity but also with diabetes, dyslipidemia, hypertension, and atherosclerosis and is considered a hepatic phenotype of lifestyle-related diseases [1–3]. Fatty liver disease without drinking habits was previously called non-alcoholic fatty liver disease (NAFLD) but was renamed metabolic dysfunction-associated steatotic liver disease (MASLD) in 2023 [4]. With the name change from NAFLD to MASLD, the diagnostic criteria clearly stated that one or more of the five cardiometabolic criteria (obesity, hypertension, diabetes, low-density lipoprotein (LDL) cholesterol, high-density lipoprotein (HDL) cholesterol, and high triglycerides) must be met, making it more closely related to lifestyle-related diseases. MASLD, a lifestyle-related disease, is on the rise worldwide, with a prevalence of 30% [5].

Recently, genome-wide association studies (GWAS) have identified many single nucleotide polymorphisms (SNPs) associated with fatty liver disease. In 2008, Romeo reported the patatin-like phospholipase domain-containing 3 (PNPLA3) gene [6]. PNPLA3 rs738409 (C > G) is associated with NAFLD in many ethnic groups, including the Japanese [7–10]. Besides PNPLA3, various other SNPs have been reported to be associated with NAFLD [11–19]. Among the many NAFLD-related SNPs, the PNPLA3 rs738409 SNP (C > G) is common in Japan, and the overall Japanese prevalence of the GG genotype is reported to be approximately 20% and 40% in patients with NAFLD [20–22].

The gut microbiota is deeply involved in the development of liver fat and fibrosis, and the association between the gut microbiota and the liver is referred to as the gut-liver axis [23]. Many studies have investigated the relationship between gut microbiota and fatty liver [24, 25]. Moreover, recent studies have indicated a link between host genes and the gut microbiota [26–29]. Studies examining this association have been limited and have focused primarily on Western populations. However, a recent study on the Japanese individuals revealed that host genetic factors, including SNPs, have a significant impact on the composition and function of the gut microbiota [30, 31].

Although many cross-sectional studies have investigated the relationship between PNPLA3 rs738409, fatty liver, and fibrosis, few cohort studies have been conducted in healthy individuals without fatty liver. Furthermore, few epidemiological studies have investigated the relationship between PNPLA3 rs738409 and the gut microbiota in liver fat and fibrosis progression. Studying the effects of gene polymorphisms on the progression of liver fat and fibrosis in healthy non-fatty liver over time in the same population is important for preventing the development of MASLD, a lifestyle-related disease, and extending healthy life expectancy. This study investigated the effects of PNPLA3 rs738409 on liver fat and fibrosis over time in a general population without fatty liver disease, including its involvement in the gut microbiota.

## Material and methods

### Study subjects

**Fig. 1:**
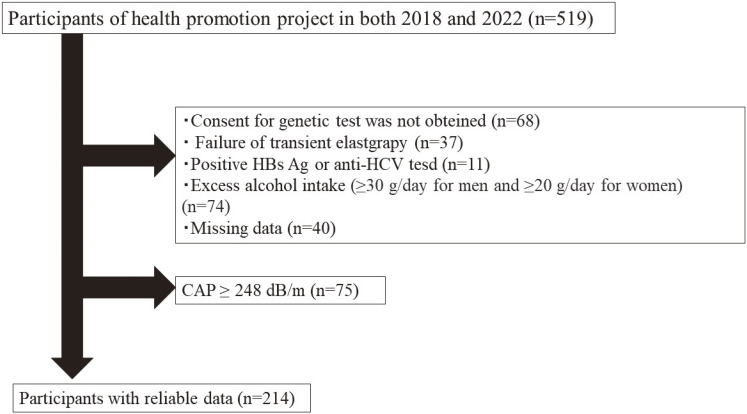
Study enrollment flowchart.

This study was conducted as part of the Iwaki Health Promotion Project, a community-based health promotion project targeting the general Japanese population. The Iwaki Project is conducted every June as a regular health checkup for residents of the Iwaki area of Hirosaki City, Aomori Prefecture [32]. All participants were adults (19–88 years old) who voluntarily responded to the open calls. There were 519 adult participants in the Iwaki Health Promotion Projects held in June 2018 and June 2022 in the Iwaki District of Hirosaki City, northern Japan (Fig. 1). Participants who could not give consent for genetic testing, failure of transient elastography measurement, positive HBs Ag or anti-HCV, excess alcohol intake (≥30 g/day for men and ≥20 g/day for women), missing data, and controlled attenuation parameter (CAP) value of ≥248 dB/m were excluded. A total of 214 participants with non-fatty liver were included in this analysis. A calculation of sample size with an effect size of 0.25, a significant level of 5%, and a power of 95%, the required total sample size was calculated to be 184 cases. The number of subjects in this study was larger than the required sample size.

### Transient elastography

The controlled attenuation parameter (CAP) and liver stiffness measurements (LSM) were performed using a FibroScan 530 (Echosens, Paris, France) with M and XL probes. All the tests were performed by five professionally trained hepatologists. Measurements were excluded if the number of measurements was <10 or if the interquartile range ratio was >0.30 because of unreliability. In accordance with previous studies, a CAP value ≥248 dB/m was defined as liver steatosis [33].

### Clinical parameters

The following parameters were measured: age, sex, height, weight, body mass index (BMI), aspartate aminotransferase, alanine aminotransferase, gamma-glutamyl transpeptidase, fasting blood glucose, insulin, triglyceride, HDL cholesterol, LDL cholesterol, smoking, and alcohol consumption habits. The Homeostasis Model Assessment of Insulin Resistance (HOMA-IR) was calculated using the following formula: fasting blood glucose (mg/dL) × fasting insulin (µU/mL)/405.

### DNA preparation and SNP genotyping

SNP genotypes were determined by whole-genome sequencing with imputation from the Japonica Array (Toshiba, Tokyo, Japan), which comprises population-specific SNP markers designed from 1070 whole-genome reference panels and TaqMan PCR [34, 35]. Whole-genome sequencing and imputation were performed by Takara Bio Corporation (Shiga, Japan) and Toshiba Corporation (Tokyo, Japan), respectively. For the Japonica Array, DNA was purified from peripheral whole blood using a QIAamp.^®^ 96 DNA Blood Kit (QIAGEN, Hilden, Germany) and extracted from plasma pellets for whole-genome sequencing. Among the many SNPs extracted by the Japonica Array, this study focused on SNP PNPLA3 rs738409, which has been reported to be most involved in the onset and progression of MASLD in previous studies [7, 8, 10, 20].

### Measurements of the gut microbiota

The gut microbiota data were obtained using the following procedure: The participants were provided with a fecal sample kit in advance, and fecal samples were collected at home. DNA was extracted from the bead-beaten fecal suspensions using an automated nucleic acid extraction system (Precision System Science). A MagDEA DNA 200 (GC) reagent kit (Precision System Science) was used for automated nucleic acid extraction. DNA extraction from all samples was completed within 4 months. Universal primer sets were used to amplify the V3-V4 regions of the 16S rRNA gene. Solution preparation and condition setting for PCR amplification were performed as described previously [36]. PCR fragments purified using PCR Cleanup Filter Plates (Merck Millipore, Burlington, MA, USA) were quantified by real-time quantitative PCR (qPCR). To read DNA sequences, purified PCR fragments were analyzed by paired-end sequencing of 2 × 300 cycles on a MiSeq™ system (Illumina, San Diego, CA, USA). Paired-end reads were processed as follows: adapter sequences and low-quality bases (Q < 20) at the 3′ end of the reads were trimmed using Cutadapt (version: 1.13). Reads containing ambiguous bases N or shorter than 150 bp were excluded. Paired-end reads that met the criteria were merged into a single read called a “merged read.” Merged reads shorter than 370 bp or longer than 470 bp were excluded using the fastq_mergepairs subcommand in VSEARCH (version 2.4.3) [37]. Merged reads containing one or more identified sequencing errors were excluded. After removing the chimeric reads detected using the uchime_denovo subcommand of VSEARCH, the remaining merged reads were clustered with a minimum sequence similarity of 97% to obtain operational taxonomic units (OTUs). Phylogenetic classification of OTUs was performed using the RDP classifier (commit hash: 701e229dde7cbe53d4261301e23459d91615999d) based on representative reads [38]. Predictions with a confidence score below 0.8 were treated as unclassified. The relative abundance of each bacterial genus in the gut microbiota was calculated by dividing the read count of each genus by the total read count. Of the bacterial species measured in 2018 and 2022, 386 species that were commonly extracted in both years were included in the analysis.

### Statistical analysis

Since all data analyzed followed a non-normal distribution, nonparametric tests were employed. For cases where parametric tests were required, data were natural log-transformed to approximate a normal distribution before analysis. Categorical variables are presented as frequencies and continuous variables as medians and interquartile ranges. Kruskal–Wallis and chi-square tests were used to compare the PNPLA3 genotypes. Changes from 2018 to 2022 were analyzed using the Wilcoxon signed-rank test. The interaction of PNPLA3 genotypes with changes over time from 2018 to 2022 was examined using a two-way repeated-measures analysis after the natural log transformation of the measurements to approximate a normal distribution. The microbiota were compared using the linear discriminant analysis effect size (LEfSe) [39]. The Linear Discriminant Analysis (LDA) was performed using one-against-all criteria. The LDA score threshold was 2, and the alpha value was 0.2 for pairwise Wilcoxon, respectively. Statistical analyses were performed using R software (R Foundation for Statistical Computing, version R-4.1.1) and Statistical Package for the Social Sciences (SPSS) version 28.0 (SPSS Inc., Chicago, IL, USA). Statistical significance was set at P < 0.05.

## Results

### Participants’ characteristics

The baseline participant characteristics are shown in Table 1. Of the participants, 68 (31.8%) had the PNPLA3 CC genotype, 101 (47.2%) had the CG genotype, and 45 (21.0%) had the GG genotype. There were no significant differences in age, sex, body composition, lifestyle habits, blood test results, CAP values, or LSM values among the genotypes.

**Table 1:** The characteristics of the participants

**Variables**	**CC**	**CG**	**GG**	**p-value**
	**n = 68**	**n = 101**	**n = 45**	
Age (year)	56.5 (44.5–63.8)	52.0 (38.0–64.0)	51.0 (36.5–62.0)	0.256
Sex, male	20 (29.4%)	38 (37.6%)	15 (33.3%)	0.539
BMI (kg/m^2^)	21.0 (19.1–22.8)	21.7 (19.8–23.5)	20.8 (19.4–23.1)	0.153
smoking habit	5 (7.4%)	12 (11.9%)	5 (11.1%)	0.623
exercise habit	13 (19.1%)	13 (12.9%)	8 (17.8%)	0.512
Aspartate aminotransferase (IU/L)	21.0 (17.0–25.0)	19.0 (17.0–23.0)	21.0 (17.0–26.0)	0.125
Alanine aminotransferase (IU/L)	16.0 (13.0–20.0)	15.0 (12.0–21.0)	17.0 (12.0–24.0)	0.756
γ-Glutamyl Transpeptidase (IU/L)	18.0 (15.0–27.8)	19.0 (14.0–30.5)	18.0 (13.5–27.0)	0.858
Fasting blood sugar (mg/dL)	90.0 (85.0–95.8)	89.0 (84.0–96.5)	86.0 (82.5–92.0)	0.083
HOMA-IR	0.99 (0.72–1.22)	1.01 (0.80–1.39)	0.90 (0.67–1.44)	0.452
Triglycerides (mg/dL)	65.0 (50.0–104.8)	69.0 (50.0–101.0)	67.0 (50.0–91.5)	0.892
HDL cholesterol (mg/dL)	67.0 (55.0–81.8)	63.0 (53.5–80.0)	65.0 (55.5–75.0)	0.587
LDL cholesterol (mg/dL)	118.5 (100.0–133.0)	113.0 (92.0–134.0)	105.0 (88.0–128.5)	0.277
Medication of hypertension	23 (33.8%)	21 (20.8%)	7 (15.6%)	0.051
Medication of diabetes mellitus	5 (7.4%)	5 (5.0%)	0 (0.0%)	0.190
Taking dyslipidemia	13 (19.1%)	9 (8.9%)	3 (6.7%)	0.064
CAP (dB/m)	202.0 (164.5–222.5)	199.0 (180.5–223.5)	191.0 (160.0–225.0)	0.722
LSM (kPa)	4.45 (3.50–5.48)	4.00 (3.45–4.90)	4.10 (3.15–5.10)	0.544

Data are presented as numbers (%) or median (range). BMI, body mass index; HDL, high density lipoprotein; LDL, low density; CAP, controlled attenuation parameter; LSM, liver stiffness measure

**Fig. 2:**
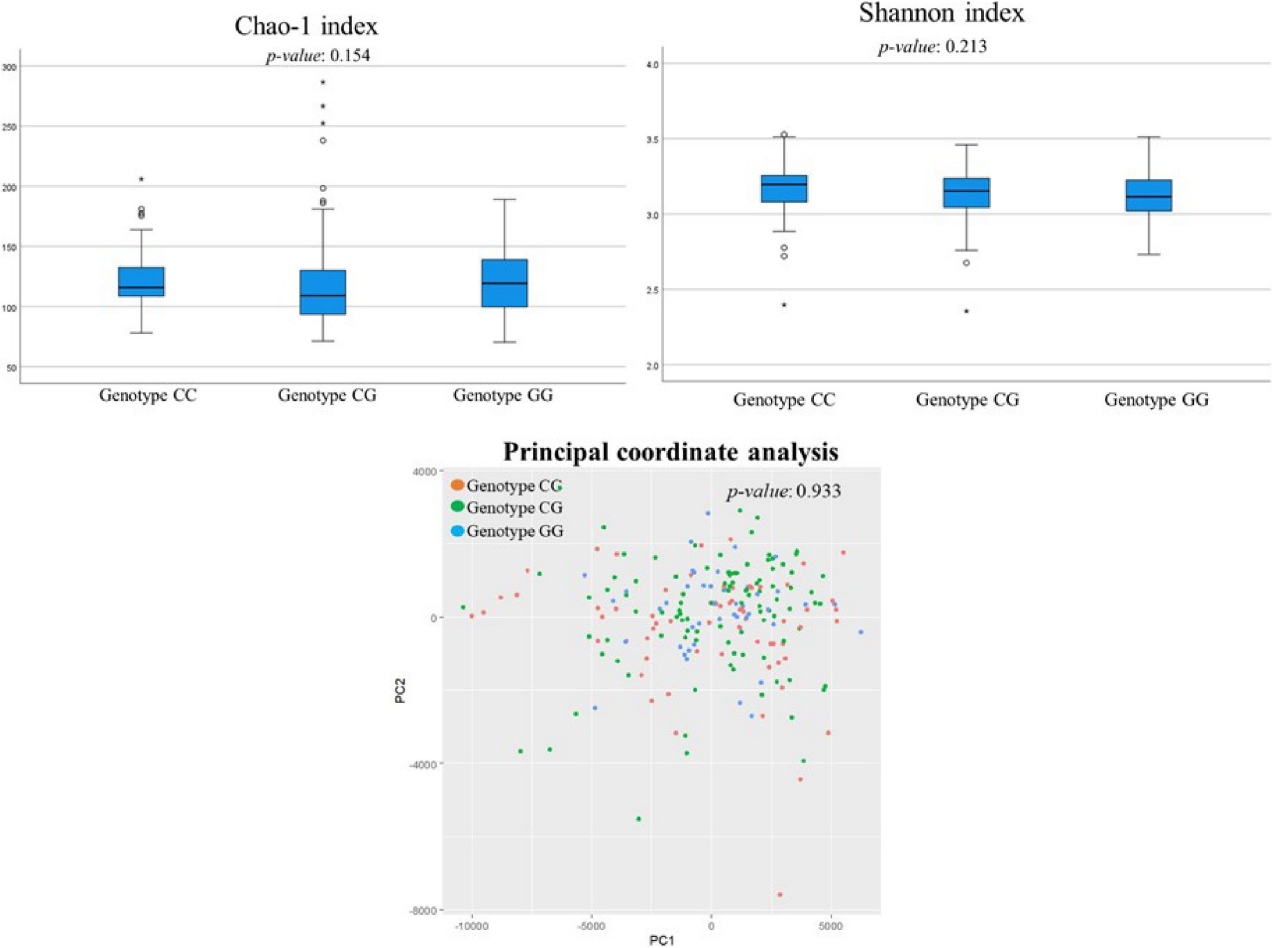
Comparison of the diversity of gut microbiota in the PNPLA3 rs738409 SNP groups.

Figure 2 shows the diversity of gut microbiota. Neither α diversity (as measured by the chao-1 and shannon indexes) nor β diversity (as assessed by principal coordinate analysis) showed significant differences across the PNPLA3 rs738409 SNP groups.

### Changes in liver fat, fibrosis, and related items from 2018 to 2022

The changes in the measurements from 2018 to 2022 are listed in Table 2. CAP values and BMI were significantly increased in individuals with CG and GG genotypes. Furthermore, the degree of increase in CAP values over four years was similar for both the CG and GG genotypes at approximately 17–18 dB/m. In 2022, 14 individuals (20.6%) with the CC genotype, 26 individuals (25.7%) with the CG genotype, and 11 individuals (24.4%) with the GG genotype showed CAP values ≥248 dB/m. LSM significantly increased in all three genotypes, but the degree of change was in the order GG (1.0 kPa) > CG (0.7 kPa) > CC (0.05 kPa). Fasting blood glucose levels increased in all genotypes; however, HOMA-IR increased significantly only in the GG genotype. HDL and LDL cholesterol levels significantly increased in all genotypes, whereas triglycerides did not show any significant changes in any genotype. Regarding liver enzymes, aspartate aminotransferase and alanine aminotransferase levels significantly increased only in the CG genotype. In contrast, no interaction was observed between the PNPLA3 genotype and changes over time.

**Table 2:** Change in liver fat, fibrosis, and related items from 2018 to 2022

**Variables**	**CC**			**CG**			**GG**			**PNPLA3×time interaction**
	**n = 68**			**n = 101**			**n = 45**			
	**2018**	**2022**	**p-value**	**2018**	**2022**	**p-value**	**2018**	**2022**	**p-value**	**p-value**
CAP (dB/m)	202.0 (164.5–222.5)	201.0 (172.5–240.5)	0.057	199.0 (180.5–223.5)	217.0 (180.0–248.0)	<0.001	191.0 (160.0–225.0)	208.0 (166.5–247.8)	0.044	0.921
LSM (kPa)	4.45 (3.50–5.48)	4.50 (3.63–6.29)	0.044	4.00 (3.45–4.90)	4.70 (4.00–5.63)	0.004	4.10 (3.15–5.10)	5.10 (3.80–5.85)	0.029	0.685
BMI (kg/m^2^)	21.0 (19.1–22.8)	21.5 (19.4–23.2)	0.074	21.7 (19.8–23.5)	22.2 (20.3–24.3)	<0.001	20.8 (19.4–23.1)	21.9 (19.8–23.4)	0.000	0.486
Fasting blood sugar (mg/dL)	90.0 (85.0–95.8)	94.5 (90.0–102.0)	<0.001	89.0 (84.0–96.5)	95.0 (88.5–104.0)	<0.001	86.0 (82.5–92.0)	91.0 (88.0–98.0)	<0.001	0.927
HOMA-IR	0.99 (0.72–1.22)	1.03 (0.76–1.51)	0.065	1.01 (0.80–1.39)	1.06 (0.78–1.44)	0.661	0.90 (0.67–1.44)	1.12 (0.75–1.79)	0.002	0.136
Triglycerides (mg/dL)	65.0 (50.0–104.8)	67.5 (47.5–97.0)	0.387	69.0 (50.0–101.0)	71.0 (50.5–109.0)	0.302	67.0 (50.5–91.5)	75.0 (50.0–95.5)	0.243	0.408
HDL cholesterol (mg/dL)	67.0 (55.0–81.8)	75.0 (63.0–88.5)	<0.001	63.0 (53.5–80.0)	70.0 (59.0–84.5)	<0.001	65.0 (55.5–75.0)	74.0 (59.5–88.5)	<0.001	0.889
LDL cholesterol (mg/dL)	118.5 (100.0–133.0)	127.5 (101.8–144.8)	0.005	113.0 (92.0–134.0)	118.0 (101.0–146.0)	<0.001	105.0 (88.0–128.5)	120.0 (102.5–135.5)	<0.001	0.704
Aspartate aminotransferase (IU/L)	21.0 (17.0–25.0)	21.0 (18.3–25.0)	0.165	19.0 (17.0–23.0)	20.0 (18.5–23.5)	<0.001	21.0 (17.0–26.0)	20.0 (18.0–25.0)	0.269	0.393
Alanine aminotransferase (IU/L)	16.0 (13.0–20.0)	16.5 (14.0–20.0)	0.575	15.0 (12.0–21.0)	18.0 (14.0–22.0)	0.022	17.0 (12.0–24.0)	18.0 (13.0–25.0)	0.800	0.372
γ-Glutamyl TransPeptidase (IU/L)	18.0 (15.0–27.8)	19.0 (15.3–27.8)	0.285	19.0 (14.0–30.5)	20.0 (15.0–34.0)	0.287	18.0 (13.5–27.0)	18.0 (14.0–29.5)	0.441	0.241

Data are presented as median (range). BMI, body mass index; HDL, high density lipoprotein; LDL, low density; CAP, controlled attenuation parameter; LSM, liver stiffness measure

### Changes in gut bacteria species from 2018 to 2022

**Fig. 3:**
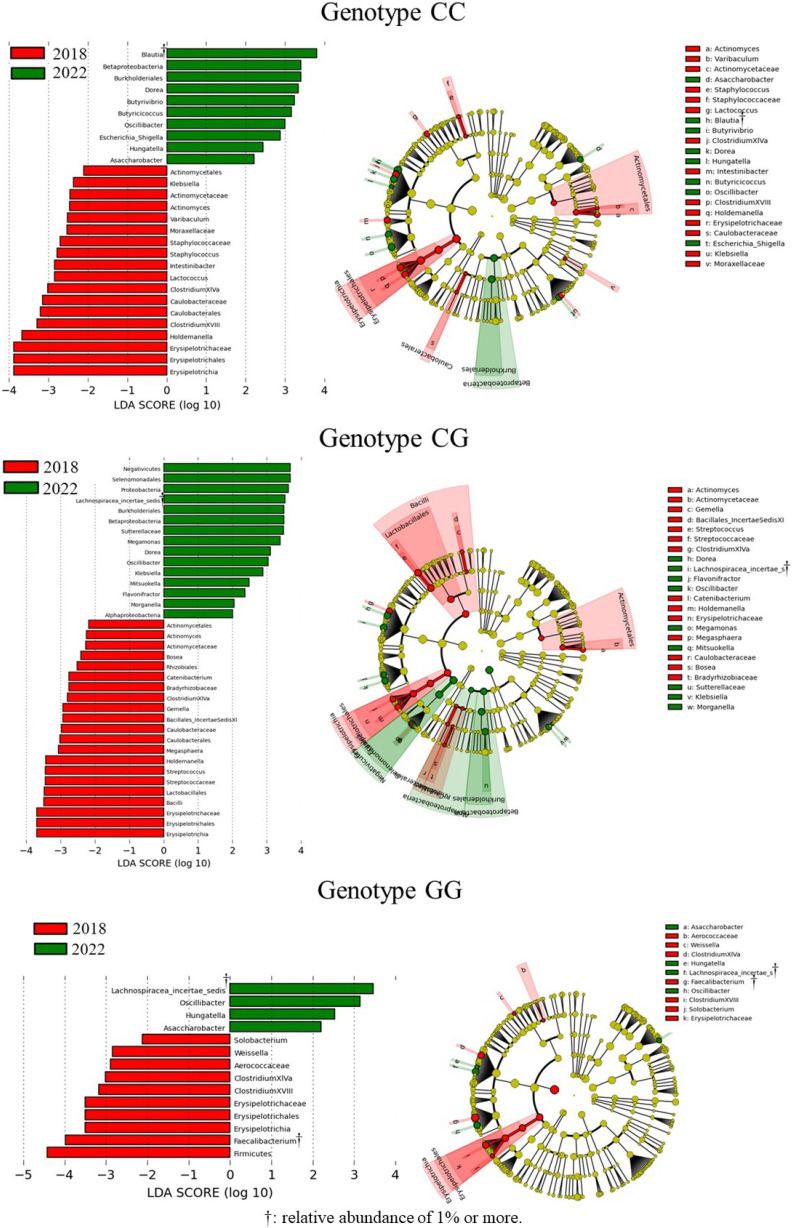
The LEfSe results of the gut microbiota between 2018 and 2022 by PNPLA3 SNPs.

**Fig. 4:**
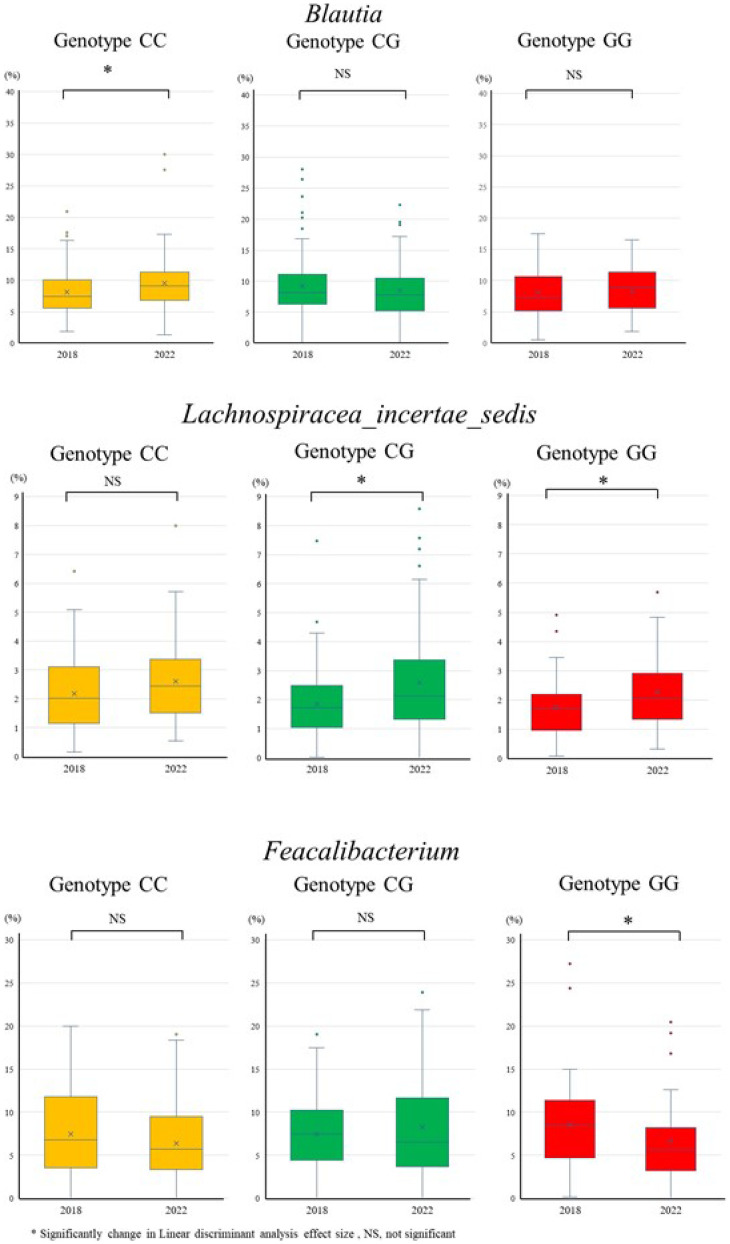
Change in gut microbiota relative abundance from 2018 to 2022.

The results of the LEfSe analysis of changes in the gut microbiota species from 2018 to 2022 are shown in Fig. 3. Twenty-eight species of the genotype CC, 36 species of the genotype CG, and 14 species of the genotype GG exhibited significant changes. Of these, the genera with subclasses with relative abundances greater than 1% were *Blautia*, which increased only in genotype CC; *Feacalibacterium*, which decreased only in genotype GG; *Lachnospiracea_incertae_sedis*, which increased in genotypes CG and GG (Fig. 4).

**Fig. 5:**
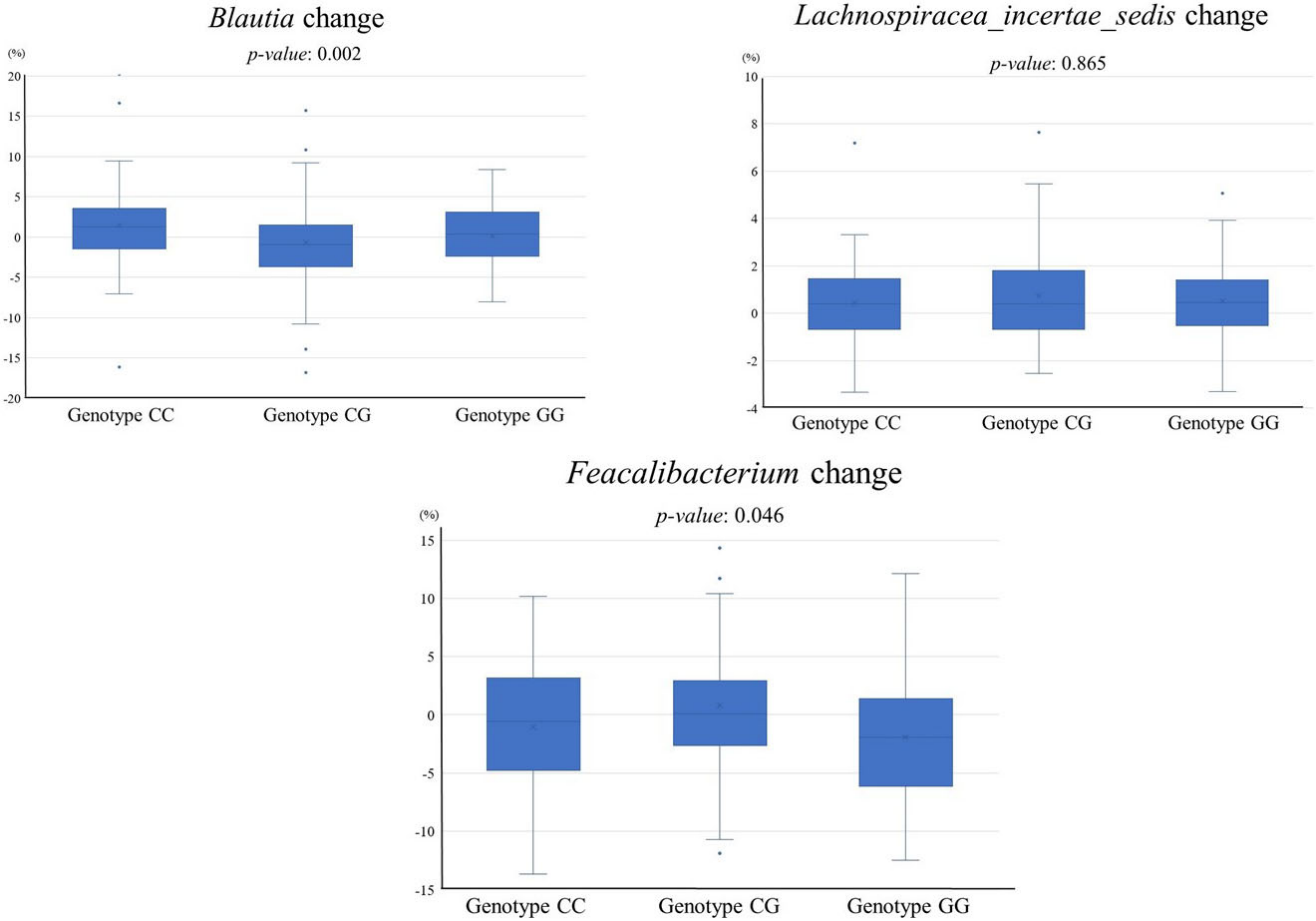
Comparison of changes in gut microbiota relative abundance from 2018 to 2022 by PNPLA3 genotype.

The amounts of change in the relative abundances of *Blautia*, *Lachnospiracea_incertae_sedis*, and *Feacalibacterium* by PNPLA3 rs738409 genotypes from 2018 to 2022 were investigated (Fig. 5). Significant differences were observed between *Blautia* and *Feacalibacterium*. Although *Feacalibacterium* decreased in all genotypes over the study period, with the GG genotype showing a greater decrease of >2% compared with the CC and CG genotypes. *Blautia* significantly increased in the CC genotype. The amounts of change in *Blautia* differed among the PNPLA3 rs738409 genotypes, but there was no consistent trend.

## Discussion

Our study is a landmark study that differs from other previous studies that have longitudinally studied the effects of PNPLA3 rs738409 on liver fat and fibrosis, including its involvement in the gut microbiota. In this study, in the general population without fatty liver, the PNPLA3 rs738409 GG and CG genotypes were associated with increased liver fat mass over 4 years compared to the CC genotype. Furthermore, all PNPLA3 rs738409 genotypes showed progression of liver fibrosis over 4 years; however, genotype GG showed a greater degree of liver fibrosis progression and worse insulin resistance than the other genotypes. The PNPLA3 rs738409 GG genotype also revealed a 2-fold decrease in gut *Feacalibacterium* over a 4-year period compared to the CC and CG genotypes. While there was an association over a longer course of four years, there was no difference among the PNPLA3 rs738409 genotypes at baseline.

Among the study participants in the general population with non-fatty liver, the GG genotype of PNPLA rs738409 was 21.0%. The prevalence of the PNPLA3 rs738409 GG genotype in Japan is estimated to be 20% in the total population and 40% in patients with NAFLD [20–22]. Although there are differences in diagnostic methods, such as the use of liver biopsy for fatty liver in previous studies, whereas FibroScan was used in this study, the study participants were generally consistent with those of previous studies.

In this study, the PNPLA3 rs738409 GG genotype was associated with worse liver fat mass, liver fibrosis, and insulin resistance than the other genotypes. In a study of Japanese participants, the GG genotype of PNPLA3 rs738409 was reported to cause more advanced fibrosis and liver-related diseases than the other genotypes [40]. The G-allele carrier of PNPLA3 rs738409 causes hepatic lipolysis by inhibiting the function of other lipases that compete with the cofactor 1-acylglycerol-3-phosphate O-acyltransferase (CGI-58/ABDH5), especially adipose triglyceride lipase (ATGL) encoded in PNPLA2, which is important for lipolysis [41]. Furthermore, carriers of the PNPLA3 G allele show increased fibrosis due to the loss of retinyl-palmitate lipase activity in hepatic astrocytes and inhibition of retinol production, which suppresses liver fibrosis [42, 43].

In this study, the PNPLA3 rs738409 GG genotype also showed significantly increased BMI and HOMA-IR over 4 years. PNPLA3 rs738409 G allele carriers reportedly exhibit worse insulin resistance [44, 45]. The PNPLA3 rs738409 genotype GG might promote liver fat and fibrosis by worsening insulin resistance, in addition to direct hepatic fattening and fibrosis effects, compared to other genotypes. Previous studies have reported that the PNPLA3 rs738409 GG genotype is associated with lower BMI and lean NAFLD than other genotypes [46, 47]. However, this study differs from previous studies in that there was no difference in BMI between PNPLA3 genotypes at baseline, but BMI increased significantly in PNPLA3 rs738409 G allele carriers over 4 years. The previous study was cross-sectional, whereas this was a cohort study of the same individuals over time. Moreover, baseline comparisons of the study participants showed no differences among the PNPLA3 rs738409 genotypes in CAP levels as well as items related to obesity, such as blood glucose, cholesterol, and exercise habits. SNPs including fat mass and obesity-associated (FTO), brain-derived neurotrophic factor (BDNF), and melanocortin 4 receptor (MC4R) have also been implicated in obesity in the Japanese population [48–50]. Because of these factors, the study may not have found a difference in BMI between baseline genotypes. The PNPLA3 rs738409 GG genotype significantly increased not only cholesterol but also HOMA-IR over 4 years, suggesting that the increase in BMI may have been caused by worsening insulin resistance.

In this study, gut *Feacalibacterium* was significantly decreased in the PNPLA3 rs738409 GG genotype group compared to that in the CC and CG genotype groups. *Faecalibacterium* is the major butyric acid-producing bacterium, and is reduced in inflammatory bowel disease and MASLD [51, 52]. Butyric acid suppresses intestinal permeability and inflammation via regulatory T cells, thereby reducing the influx of toxic substances, including endotoxin, into the liver, and the administration of butyrate-producing bacteria prevents liver dysfunction [53–55]. Furthermore, a previous study on Japanese individuals investigating the relationship between host genetic factors and gut microbiota identified *Clostridiales*, *Ruminococcaceae*, *Erysipelotrichaceae*, *Lachnospiraceae*, *Feacalibacterium*, and *Ruminococcus* as being influenced by genetic factors [31]. We previously reported that the composition of the gut microbiota varied between healthy individuals and MASLD patients according to their PNPLA3 genotypes [56]. Although the causal relationship is unclear, this study suggests that a decrease in gut *Feacalibacterium* may be involved in the progression of liver fat and fibrosis in the PNLPA3 rs738409 GG genotype.

Among the study participants, gut *Blautia* significantly increased in the PNPLA rs738409 CC genotype over a 4-year period. *Blautia* is more common in Japan and *Blautia* has been reported to increase acetate production and suppress NAFLD/NASH [57, 58]. In fact, NAFLD/NASH patients have reduced *Balutia* [59, 60]. Unlike the CG and GG groups, the PNPLA3 rs738409 CC genotype group in this study showed no increase in CAP values or BMI from 2018 to 2022, which could be due to increased gut *Blautia*. However, other studies in Japanese populations have reported that *Blautia* is not affected by host genetics [31]. Although both studies targeted Japanese populations, differences in the age, residence, and lifestyle of the subjects may have contributed to the discrepancy in the results.

*Lachnospiracea_incertae_sedis* is considered one of the butyrate-producing bacteria that are important for maintaining intestinal homeostasis [61, 62]. *Lachnospiracea_incertae_sedis* has been linked to autoimmune diseases such as Crohn’s disease, dementia, and autism spectrum disorders; however, unlike *Feacalibacerium*, which is also a butyrate-producing bacterium, its specific function is unknown [63–66]. *Lachnospiracea_incertae_sedis* significantly increased over 4 years in both PNPLA3 rs738409 CG and GG genotypes in this study, but the specific association between liver fat and fibrosis remains unclear and requires further investigation [53].

Findings on the association between PNPLA3 rs738409 and gut microbiota are inconsistent, with reports that there is no association and the bacterial species involved differing from study to study [67–69]. Previous studies have focused on young, lean, or obese MASLD subjects, whereas our study focused on relatively healthy general population, mainly middle-aged subjects. Furthermore, the mechanisms by which PNPLA3 rs738409 and other host genetic factors affect gut bacteria are not fully elucidated [68, 69]. Environmental factors such as diet, lifestyle, and medications may mitigate or obscure the effects of the host’s genetic factors on the gut microbiota [70, 71]. This study is also valuable in terms of a cohort study, but has some drawbacks, such as the sample size is not that large, the focus is on middle-aged subjects of one small region of Japan. Large-scale cohort studies have been suggested to be necessary to further elucidate the mechanisms by which PNPLA3 rs738409 influences gut microbiota [67].

In this study, PNPLA3 rs738409 CC genotype showed no significant change in CAP values over 4 years, but, 20% of individuals with the PNPLA3 rs738409 CC genotype had CAP values exceeding 248 dB/m, the cutoff for fatty liver, in 2022. In the PNPLA3 rs738409 CC genotype group, CAP values were positively correlated with age (correlation coefficient 0.252, p = 0.038, data not shown), and participants with CAP values of 248 dB/m or higher in 2022 were 2.5 years older than participants with CAP values of <248 dB/m. In addition, LDL cholesterol and fasting blood glucose levels also increased in the PNPLA3 rs738409 CC genotype group over the 4-year period. Even in individuals with the PNPLA3 rs738409 CC genotype, long-term exposure to aging, hyperglycemia, and dyslipidemia may lead to fatty liver development.

In this study, there were no significant differences among the genotypes at baseline. Although genetics play a role in liver fat and fibrosis, numerous other factors contribute to its development. The multiple parallel hit hypothesis posits that organs beyond the liver, including adipose tissue, oral cavity, and intestines, work together in a complex manner to influence disease progression [72]. Furthermore, many SNPs besides PNPLA3 rs738409 are associated with liver fat and fibrosis [11–19, 22]. SNPs other than PNPLA3 rs738409 or other factors, such as diet and lifestyle, may have contributed to the lack of association among the PNPLA3 rs738409 genotypes at baseline. However, a significant association with the PNPLA3 rs738409 genotype was observed at 4 years, suggesting that the effect of PNPLA3 rs730409 on liver fat and fibrosis requires a yearly course. On the other hand, it was possible that changes in diet, lifestyle, and oral medications over the four years could have caused the changes observed over time, and therefore caution should be exercised in interpreting the results.

This study has several limitations. First, the subjects of this study were limited to the Iwaki area of Hirosaki City, Aomori Prefecture, Japan. Because gut microbiota and genetic polymorphisms are highly dependent on diet, lifestyle and genetic background, the results of this study cannot be applied to all ethnic groups. Therefore, the results of this study cannot be generalized to all ethnic groups. Second, although this study investigated the gut microbiota, it was influenced by various factors, such as diet and medication. As this study did not sufficiently adjust for these confounding factors, caution is required when interpreting the results. Third, liver fat and fibrosis were diagnosed using a FibroScan as an alternative to liver biopsy. Invasive liver biopsy, which is commonly performed as part of a health checkup in the general population, was not feasible in this study. Fourth, while this study found a correlation between PNPLA3 gene polymorphisms and intestinal bacteria, a causal relationship has not been elucidated. Clarification of the causal relationship is an important issue to be resolved in the future.

## Conclusions

This cohort study revealed that PNPLA3 rs738409 G-allele carriers had significantly more advanced liver fat and fibrosis than noncarriers, even in non-fatty liver population. The mechanism by which PNPLA3 rs738409 G-allele carriers promote liver fat and fibrosis, in addition to their direct effects on hepatocytes or through insulin resistance and obesity, the effects via decrease of gut *Feacalibacterium* were suggested. However, as the PNPLA3 rs738408 SNP has a relatively long-lasting effect on liver fat and fibrosis, lifestyle modifications such as diet and exercise may prevent liver fat and fibrosis in the future. Since MASLD is a hepatic phenotype of lifestyle-related diseases, lifestyle modifications such as diet and exercise are important for its prevention. On the other hand, this study suggests that even with the same lifestyle, the onset and progression of MASLD varies depending on the genetic background, and the involvement of gut microbiota as a factor in this process. In the prevention of MASLD, personalized medicine such as administration of prebiotics based on PNPLA3 gene polymorphisms may be more important. On the other hand, the causal relationship between genetic polymorphisms and gut microbiota has not been fully elucidated in this study or in previous studies. Future research, particularly large-scale cohort studies encompassing various ethnicities, age groups, and geographic locations, is necessary to elucidate this complex relationship.

## Abbreviations

NAFLD: nonalcoholic fatty liver disease
MASLD: metabolic dysfunction-associated steatotic liver disease
SNP: single nucleotide polymorphism
PNPLA3: patatin-like phospholipase domain-containing 3
CAP: controlled attenuation parameter
LSM: liver stiffness measurement

## Declarations

### Availability of data and materials

Data presented in this study are available upon request from the corresponding author. The data are not publicly available due to privacy and ethical restrictions.
